# SHP2 Inhibitors Show Anti-Myeloma Activity and Synergize With Bortezomib in the Treatment of Multiple Myeloma

**DOI:** 10.3389/fphar.2022.841308

**Published:** 2022-04-06

**Authors:** Pan Zhou, Mengyu Xiao, Weiya Li, Xiaobai Sun, Yanliang Bai, Feiying Meng, Zunmin Zhu, Weiping Yuan, Kai Sun

**Affiliations:** ^1^ Department of Hematology, Henan Provincial People’s Hospital, Zhengzhou University People’s Hospital, Zhengzhou, China; ^2^ Henan Eye Institute, Henan Eye Hospital, Henan Provincial People’s Hospital, Zhengzhou University People’s Hospital, Zhengzhou, China; ^3^ State Key Laboratory of Experimental Hematology, Institute of Hematology and Blood Diseases Hospital, Chinese Academy of Medical Sciences and Peking Union Medical College, Tianjin, China

**Keywords:** multiple myeloma, SHP2, ERK pathway, bortezomib, synergistic effect

## Abstract

Multiple myeloma (MM) is a plasma cell malignancy that remains incurable. The protein tyrosine phosphatase SHP2 is a central node regulating RAS/mitogen-activated protein kinase (MAPK)/extracellular signal regulated kinase (ERK) signaling pathway which plays a crucial role in the pathogenesis and proteasome inhibitor (PI) resistance of MM. Several preclinical studies have demonstrated that SHP2 inhibitors exerted antitumor activity in cancer-harboring diverse mutations in the RAS pathway, offering the potential for targeting myeloma. In this study, we showed that pharmacological inhibition of SHP2 activity using SHP099 and RMC-4550 efficiently inhibited the proliferation of MM cells by inducing apoptosis and cell cycle arrest. As per the mechanism, SHP2 inhibitors activated the level of cleaved caspase3, BAK, and P21 and downregulated ERK phosphorylation in MM cells. Moreover, the blockade of SHP2 exhibited anti-myeloma effect *in vivo* in a mouse xenograft model. In addition, SHP2 inhibitors synergized the antineoplastic effect of bortezomib in bortezomib-sensitive MM cells and showed identical efficacy in targeting bortezomib-resistant MM cells. Overall, our findings suggest that SHP2-specific inhibitors trigger anti-myeloma activity *in vitro* and *in vivo* by regulating the ERK pathway and enhancing cytotoxicity of bortezomib, providing therapeutic benefits for both bortezomib naïve and resistant MM.

## 1 Introduction

Multiple myeloma is a hematologic malignancy characterized by clonal expansion of malignant plasma cells predominantly in the bone marrow and accounts for more than 10% of all hematologic cancers (Kumar et al., 2017). In the past decade, many effective anti-myeloma treatments have been developed, including proteasome inhibitors (PIs), immunomodulatory drugs (IMiDs), histone deacetylase inhibitors, monoclonal antibodies (mAbs), and chimeric antigen receptor T-cell (CAR-T) therapy, and have markedly increased overall survival (Chim et al., 2018). Nonetheless, due to the inevitable relapse or chemotherapy resistance of the disease course, MM, heretofore, has still been considered fatal and incurable (Bazarbachi et al., 2019). Therefore, there remains an urgent need to develop novel drugs that target key pathways and/or overcome drug resistance to improve the survival outcome of MM.

SH2 domain–containing tyrosine phosphatase 2 (SHP2), encoded by the PTPN11 gene, is a 593-amino acid classical non-receptor protein tyrosine phosphatase (PTP) containing two Src homology-2 domains (N-SH2, C-SH2), a PTP domain, and a carboxyl-terminal region. In the basal state, SHP2 is autoinhibited by the N-SH2 domain and PTP domain interaction, which then blocks substrate access (Chan et al., 2008; Sun et al., 2018). However, oncogenic mutations of SHP2 destabilize the autoinhibited conformation and activate enzyme activity in the absence of tyrosine-phosphorylated ligand stimulation (Larochelle et al., 2018). Recently, an oncogenic role for SHP2, which is hyperactivated due to amplification or hyperphosphorylation of SHP2-recruiting/activating proteins, has been reported during oncogenesis in many cancers, especially hematological malignancies (Zhang et al., 2015; Pandey et al., 2017). For example, SHP2 is required for the full activation of RAS/mitogen-activated protein kinase (MAPK)/extracellular signal–regulated kinase (ERK) signaling. In addition, SHP2 has also emerged as a pivotal convergent node of multiple signaling pathways such as JAK/STAT, PI3K/AKT, and PD-1/PD-L1 pathways (Song et al., 2021). Since the dysregulation of SHP2 contributes to the activation of various oncogenic signaling cascades, it makes SHP2 an attractive therapeutic target for cancer therapy. Encouragingly, the discovery of allosteric inhibitors of SHP2 featured with high potency, selection, and oral bioactivity offers an appealing and novel approach to suppress the growth of RAS-driven cancer cells, including lung cancer, pancreatic cancer, and leukemia cells (Garcia Fortanet et al., 2016; Dardaei et al., 2018; Fedele et al., 2018; Wang et al., 2020). SHP099 and RMC-4550, represented as the newly developed allosteric inhibitors of SHP2, concurrently bind to the interface of the N-terminal SH2, C-terminal SH2, and PTP domain, thus stabilizing it in an inactive conformation (Liu et al., 2020). As reported, the RAS/MAPK/ERK pathway is currently believed to be a key pathway activated in about half of the MM cases and is, therefore, considered to be a significant therapeutic target in MM (Chang-Yew Leow et al., 2013). However, the individual role of SHP2 inhibitors in treating MM remains unclear thus far.

Despite improvements in chemotherapeutic treatments, drug resistance is still a critical challenge for efficient treatment of MM during the bortezomib (BTZ)-based chemotherapy (Robak et al., 2018). Recent studies have suggested that the combination of BTZ with other drugs is one of the therapeutic strategies for overcoming BTZ resistance (Malek et al., 2017). In addition, it has been reported that MAPK pathway activation enhances PI resistance, providing a strategy for treating such patients with PI/MAPK inhibitor combinations (Shirazi et al., 2020). Moreover, the combination with SHP2 inhibitors offers an appealing approach to treat both RTK-activated and KRAS/BRAF-mutant cancers (Liu et al., 2021). Thus, there is considerable interest in the possibility that pharmacological inhibition of SHP2 may synergize with BTZ to prevent MM patients from BTZ drug resistance.

Here, we investigated the effect of SHP2 inhibitors alone and in combination with BTZ for the treatment of MM cells. Our findings revealed that SHP2 inhibition may represent a potential therapy in both BTZ naïve and resistant MM patients, and the combination treatment of SHP2 inhibitors with BTZ may be a promising therapeutic strategy for future clinical investigation.

## 2 Materials and Methods

### 2.1 Cell Culture and Development of BTZ-Resistant MM Cell Lines

RPMI-8226 and NCI-H929 were purchased from the American Type Culture Collection (ATCC, Rockville, MD, United States) and cultured in RPMI-1640 media (HyClone) supplemented with 10% fetal bovine serum (Gibco). All cells were cultured in a 37°C incubator with 5% CO_2_. BTZ-resistant RPMI-8226 and NCI-H929 cells were obtained by stepwise increasing extracellular concentrations of BTZ over a period of 9–16 months, starting at a concentration of 1 nM up to a concentration of 50 nM bortezomib.

### 2.2 Reagents and Antibodies

SHP099 (HY-100388A) and bortezomib (HY-10227) were purchased from MedChemExpress (Princeton, NJ, United States). RMC-4550 (S8718) was obtained from Selleckchem (Houston, TX, United States). The primary antibodies against SHP2 (#3397), phospho-SHP-2 (Tyr542) (#3751), and P21 (#2947) were obtained from Cell Signaling Technology. Anti-GAPDH (10494-1-AP) was purchased from Proteintech. The primary antibodies against ERK (AF1051) and phospho-ERK (AF1891) were procured from Beyotime Biotechnology. The antibodies specific for BAK (ab32371) and cleaved caspase-3 (ab32042) were obtained from Abcam. HRP-conjugated secondary antibodies were purchased from Servicebio.

### 2.3 CCK8 Assay for Cell Viability Detection

Cell viability was assessed with Cell Counting Kit-8 assay (CCK-8) (Dojindo, Kumamoto, Japan) according to the manufacturer’s instructions. The cells (1.5 × 10^4^ cells per well) were seeded into 96-well plates in a final volume of 100 μl of complete culture medium and incubated without or with small-molecule inhibitors at the indicated concentrations for 24, 48, and 72 h. Subsequently, the cells were incubated for an additional 4 h with 10 μl of CCK-8 at 37°C. The absorbance values were determined at a wavelength of 450 nm using an Absorbance Microplate Reader (SpectraMax® 190, California, United States). Drug interaction was analyzed with the Fa-CI plot, and CI calculations were performed according to the Chou–Talalay method using Calcusyn software (Biosoft, Cambridge, United Kingdom). This software determines the interaction of two drugs through calculations of the combination index (CI) based upon the multiple drug effect equation of Chou and Talalay. Denotations of CI values are as follows: >1, antagonism; 1, additivity; <1, synergy (Chou, 2010).

### 2.4 Cell Cycle Analysis by BrdU Incorporation Assay

The BrdU assay was performed using an APC-BrdU flow kit (552598, BD Bioscience Pharmingen), according to the manufacturer’s instructions. Briefly, drug-treated myeloma cells were labeled with 10 μM BrdU for 1 h. Then, the cells were fixed, permeabilized, treated with DNase (300 μg/ml for 1 h at 37°C), and stained with APC-conjugated monoclonal anti-BrdU antibody, Hoechst 33342. Finally, the cells were analyzed on a BD FACSCanto II (BD Biosciences, San Jose, CA, United States).

### 2.5 Cell Apoptosis Analysis

For apoptosis detection, the cells were harvested and stained using an Annexin V-APC/7-AAD apoptosis detection kit (BD Biosciences, San Jose, CA, United States) following the manufacturer’s protocol. FACS analysis was performed by flow cytometry using a flow cytometer (BD FACSCanto II, BD Biosciences, CA, United States) and analyzed using FlowJo (TreeStar) software.

### 2.6 Western Blot Analysis

Total protein was extracted from MM cells, and protein quantification was performed using the BCA method (Beyotime Institute of Biotechnology, Haimen, China). Subsequently, equal quantities of protein extracts (20 μg) were separated in a 10% or 12% SDS polyacrylamide gel *via* electrophoresis and transferred onto PVDF membranes (Millipore, MA, United States). Then, the membranes were blocked with 5% bovine serum albumin (BSA) for 1 h and incubated overnight at 4°C with specific primary antibodies. The blots were washed with TBS–Tween-20 (TBST) on the next day and incubated with HRP-conjugated secondary antibodies at room temperature for 1 h. The immunocomplexes were visualized using Millipore’s enhanced chemiluminescence detection system and observed in a ChemiDoc Touch Imaging System (Bio-Rad Laboratories, Inc.). GAPDH was used to normalize the amount of protein in each sample, and the relative amount of protein level was analyzed by ImageJ software.

### 2.7 Colony Formation Assay

The cells were seeded at about 500 cells/well in 24-well plates with different treatments in methylcellulose medium (MethoCult™ H4230, Stemcell Technologies, Vancouver, BC, Canada). After incubation for 7–14 days in a 5% CO_2_ atmosphere incubator at 37°C, the colonies defined as a cluster of at least 60 cells in each well were counted under an inverted phase-contrast microscope (Olympus, Japan).

### 2.8 RNA Extraction and RT-qPCR Analysis

Total RNA was extracted from cells with TRIzol reagent (Invitrogen; Thermo Fisher Scientific, Inc.) according to the manufacturer’s protocol. Reverse transcription was performed using the HiScript II Q RT SuperMix for qPCR kit according to the manufacturer’s instructions (Vazyme, Nanjing, China). Then, the mRNA expression level was measured with Universal SYBR Green Master Mixes (Roche Woburn, MA) on an ABI Prism 7500 Detection System (Applied Biosystems, Inc.). The primer pairs were as follows: BAK, forward 5′-CAT​CAA​CCG​ACG​CTA​TGA​CTC-3′ and reverse 5′- GTC​AGG​CCA​TGC​TGG​TAG​AC-3′; P21, forward5′-TGTCCGTCAGAACCCATGC-3′ and reverse 5′-AAA​GTC​GAA​GTT​CCA​TCG​CTC-3′; GAPDH, used as an internal control, forward 5′-GAC​AGT​CAG​CCG​CAT​CTT​CT-3′ and reverse 5′- TTA​AAA​GCA​GCC​CTG​GTG​AC-3′. The relative gene expression was calculated using the 2^−ΔΔCT^ method.

### 2.9 *In Vivo* Xenograft Tumor Model

Female Balb/c nude mice (aged 5 weeks) were obtained from the Beijing Vital River Laboratory Animal Technology Corporation (Beijing, China). The mice were subcutaneously injected with 5 × 10^6^ number RPMI-8226 cells in a 1:1 ratio of PBS and Matrigel basement membrane matrix (Becton Dickinson, Bedford, MA, United States). When tumors were palpable to approximately 100–130 mm^3^, tumor-bearing mice were randomly divided into two cohorts (*n* = 5) and received SHP2 inhibitors (SHP099, 75 mg/kg, p.o., qd; RMC-4550, 30 mg/kg p.o. qd) or vehicle (DMSO) for the indicated period of time. The tumor volume was estimated every 2 days and calculated using a standard formula (0.5 × length × width^2^). The mice were sacrificed by cervical dislocation at the experimental endpoint. The tumors were dissected and fixed in formalin. All the procedures used in these experiments were approved by the Guide for the Care and Use of Laboratory Animals of Zhengzhou University People’s Hospital and Henan Provincial People’s Hospital.

### 2.10 Statistical Analysis

All data shown are representative of the results of at least three independent experiments, and the results were presented as mean ± standard deviation (SD). The differences between the two groups were analyzed using Student’s t-test and one-way ANOVA followed by Dunnett’s multiple comparison test, which was used for multigroup comparisons. The data were analyzed using GraphPad prism software version 7.0 (San Diego, CA, United States). In all cases, the *p* values were designated as *p < 0.05, **p < 0.01, ***p < 0.001, and nonsignificant (*p* > 0.05).

## 3 Results

### 3.1 SHP2 Inhibitors Suppress the Cell Viability and Colony Formation Ability of MM Cells

**FIGURE 1:**
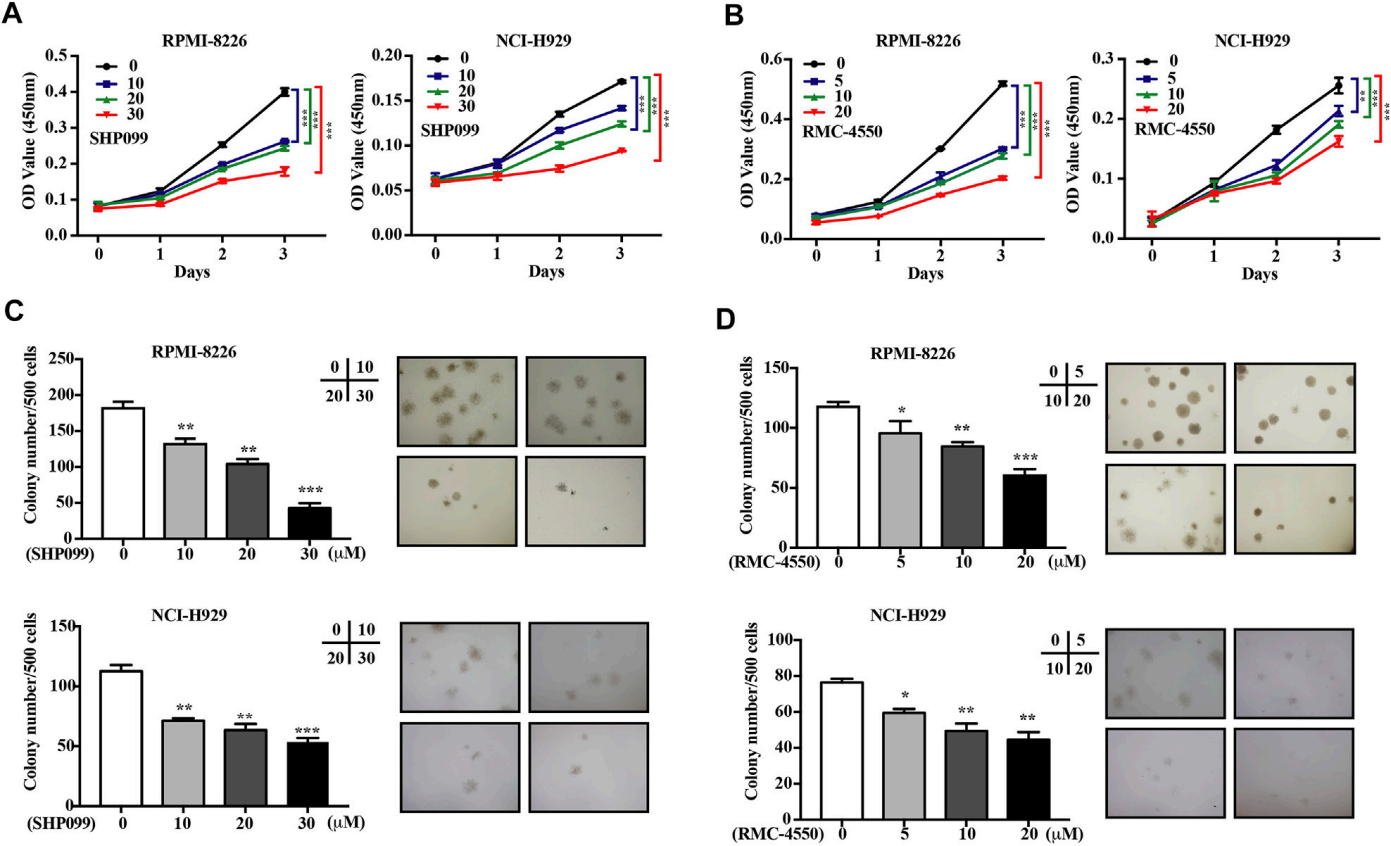
SHP2 inhibitors inhibit the cell viability and colony formation ability of MM cells *in vitro*. **(A,B)** MM cell lines (RPMI-8226 and NCI-H929) were treated with SHP099 or RMC-4550 at different concentrations for 24, 48, and 72 h, followed by measurement of the cell viability using CCK-8 assay. **(C,D)** Effect of different doses of SHP099 and RMC-4550 on the colony formation ability in MM cell lines was shown, and representative images from the surviving colonies were captured by microscopy (4×). The data are representative of the means ± SD from three independent experiments. *p < 0.05, **p < 0.01, and ***p < 0.001 vs. control.

To explore the effect of SHP2 inhibitors on MM cell viability *in vitro*, RPMI-8226 and NCI-H929 cells were treated with gradually increasing concentrations of SHP099 or RMC-4550 for 24, 48, and 72 h. The CCK-8 assay showed that SHP2 inhibitors impaired cell proliferation significantly in a dose- and time-dependent manner (Figures 1A,B). Furthermore, to evaluate the long-term survival and tumorigenic capabilities of myeloma cells exposed to SHP2 inhibitors, we then performed colony formation assays. As shown in Figures 1C,D, we observed that both colony number and size were significantly reduced by SHP099 and RMC-4550. Collectively, our results indicated that SHP2 inhibitors treatment reduced myeloma cell growth and colony formation ability.

### 3.2 SHP2 Inhibitors Provoke MM Cell Apoptosis and Induced Cell Cycle Arrest

**FIGURE 2:**
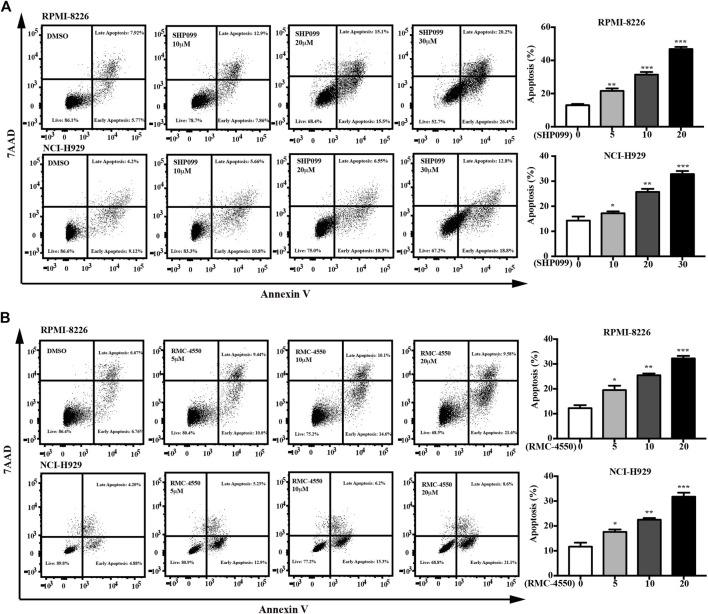
SHP2 inhibitors trigger apoptosis in MM cells. **(A)** RPMI-8226 and NCI-H929 cells were treated with 1, 10, 20, and 30 μM of SHP099 for 48 h; then, apoptotic rates were analyzed by flow cytometry. **(B)** RPMI-8226 and NCI-H929 cells were incubated with RMC-4550 (0∼20 μM) for 48 h, and apoptotic cells were detected *via* flow cytometric analyses. The results represent the mean ± SD of three independent experiments. *p < 0.05, **p < 0.01, and ***p < 0.001.

**FIGURE 3:**
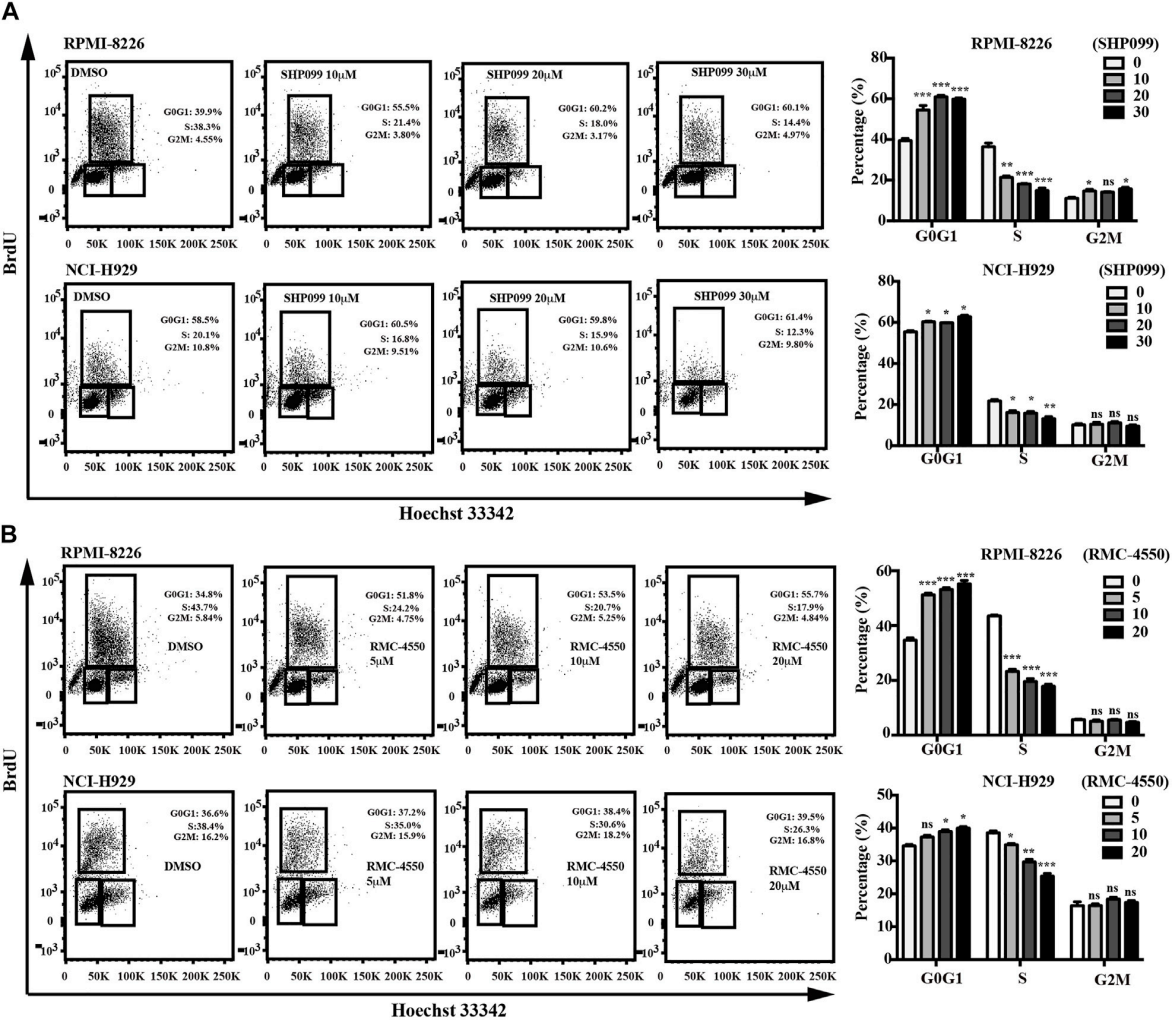
SHP2 inhibitors promote cell-cycle arrest. RPMI-8226 and NCI-H929 cells were treated with different concentrations of SHP099 **(A)** and RMC-4550 **(B)** for 48 h, and cell-cycle analysis by BrdU/Hoechst 33342 staining of MM cells was analyzed by flow cytometry. Data are representative of three individual experiments with similar results and are represented as mean ± SD. *p < 0.05, **p < 0.01, ***p < 0.001, and nonsignificant (*p* > 0.05).

Since apoptosis and cell cycle are believed to be two of the main processes that contribute to cell survival, we subsequently incubated MM cell lines with increasing concentrations of SHP2 inhibitors for 48h and then used dual staining with Annexin V-APC/7-AAD and BrdU incorporation assay by flow cytometry to examine the apoptosis-inducing and cell cycle–inhibiting effects of SHP2 inhibitors in MM cells. Our data showed increases in the percentage of apoptotic cells dose-dependently in both SHP099- and RMC-4550–treated MM cell lines (Figure 2). In addition, BrdU/Hoechst 33342 staining analysis revealed that the proportion of MM cells at G1 phase was significantly higher in cells treated with SHP099 and RMC-4550 than in those treated with DMSO, while the proportion in S phase was remarkably lower (Figure 3).

Taken together, these results suggested that SHP2 inhibitors might be potential drugs for MM treatment by inhibition of cell proliferation *via* induction of apoptosis and the arrest of cell cycle.

### 3.3 Anticancer Activity of SHP2 Inhibitors Against MM Cells is Mediated by Reducing ERK Phosphorylation

**FIGURE 4:**
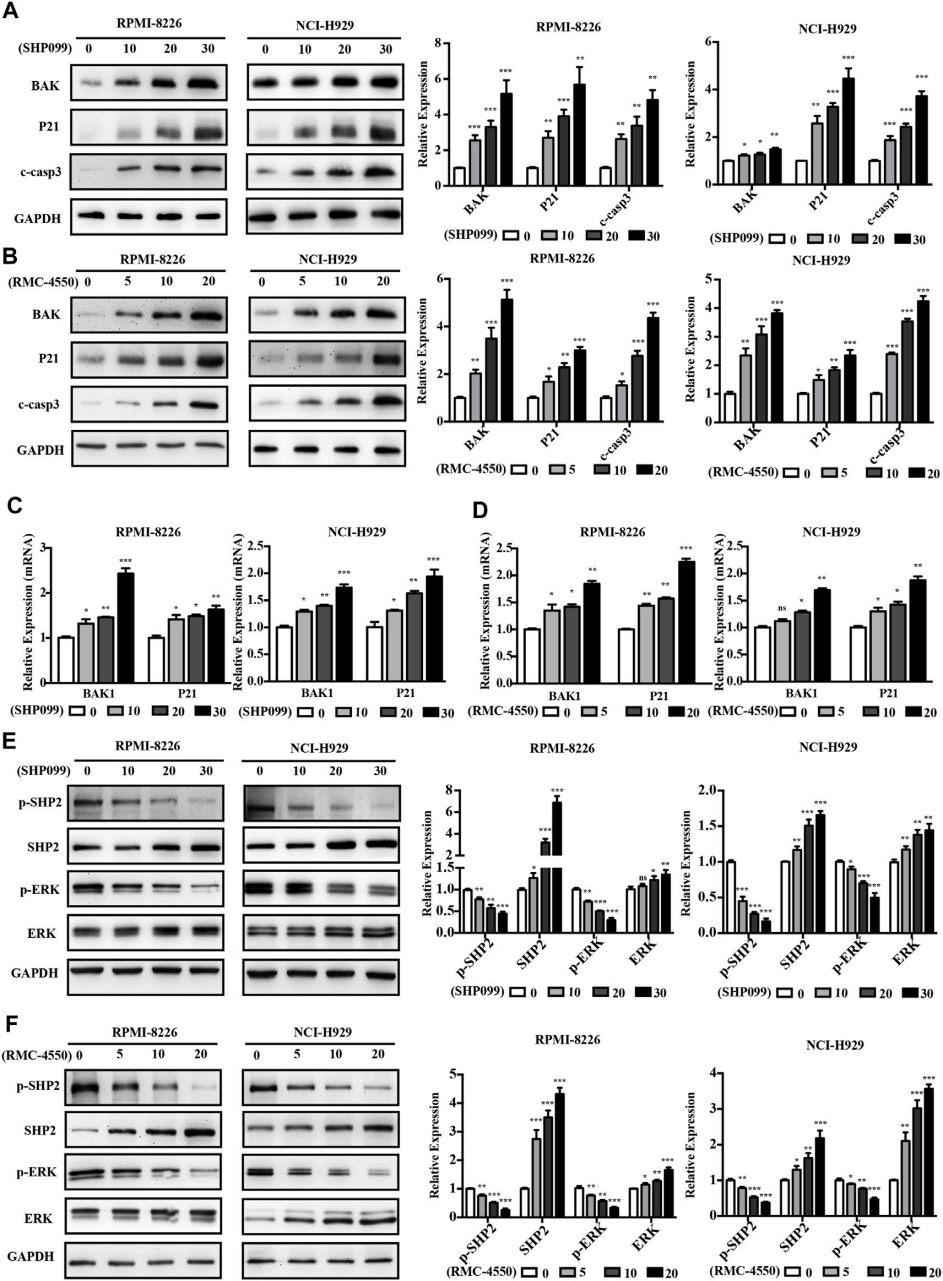
SHP2 inhibitors induce the expression of apoptosis and cell cycle–related genes and impede the activation of the SHP2/ERK pathway in MM cells. **(A,B)** Western blotting analysis showed that treatment with SHP099 or RMC-4550 for 48 h significantly enhanced cleaved caspase-3, BAK, and P21 expression in MM cells. **(C,D)** mRNA expressions of BAK and P21 were upregulated in RPMI-8226 and NCI-H929 cells after incubation with SHP099 or RMC-4550 for 48 h. **(E,F)** Expression levels of p-SHP2 and p-ERK were reduced in RPMI-8226 and NCI-H929 with SHP099 or RMC-4550. The proteins were detected using ImageJ software, and the intensity ratios of immunoblots were compared to those of the control and were quantified after normalizing with the respective loading controls for the housekeeping protein GAPDH. The data were obtained in at least three independent experiments and expressed as mean ± SD of three individual experiments. *p < 0.05, **p < 0.01, and ***p < 0.001.

To further determine the possible molecular mechanisms of cell cycle alteration and apoptosis induction mediated by SHP2 inhibitors, we used the immunoblotting analysis and found that SHP099 and RMC-4550 treatment increased the expression of apoptosis-related protein cleaved caspase-3, BAK, and cell cycle inhibitor P21 of MM cells in a concentration-dependent manner (Figures 4A,B). Meanwhile, the mRNA levels of BAK and P21 were also significantly elevated in MM cells with the exposure of the SHP2 inhibitors (Figures 4C,D). Given the role of SHP2 in the activation of the RAS/ERK pathway, we speculated that SHP2 inhibitors impede MM cell proliferation and cell-cycle progression by regulating the ERK signaling pathway. Hence, MM cells were incubated with dose-increased levels of SHP099 or RMC-4550 for 48 h and blotted proteins of the SHP2/ERK signaling pathway. Indeed, both SHP099 and RMC-4550 treatments could concentration-dependently inhibit the expression of phosphorylated SHP2 and ERK with apparent upregulation of the expression of total SHP2 and ERK (Figures 4E,F).

Collectively, the aforementioned data implied that the ERK pathway plays a crucial role in SHP2 inhibitor–mediated cell apoptosis and cell cycle arrest in MM cells.

### 3.4 SHP2 Inhibitors Attenuate Tumor Growth in Murine Xenograft Models

**FIGURE 5:**
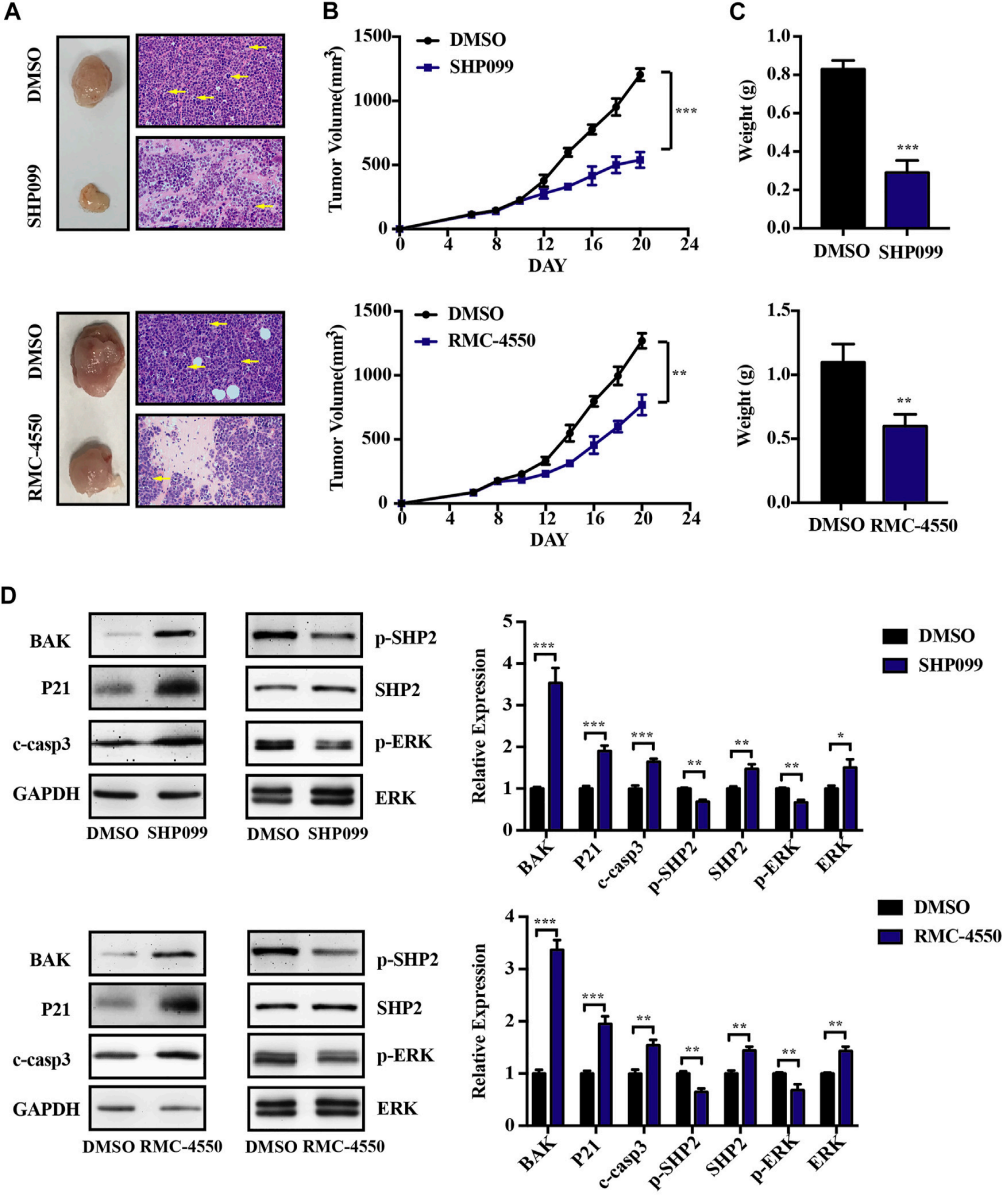
SHP2 inhibitors show antitumor effects *in vivo* by using MM xenograft mouse models. **(A)** Representative images of tumors isolated from mice after approximately 2 weeks of treatment with SHP2 inhibitors (SHP099 or RMC-4550) or vehicle and H&E staining of tumor sections were shown. The pathological mitosis was marked with yellow arrows. **(B)** Tumor volume was measured every 2 days, and tumor growth rates were significantly reduced in SHP2 inhibitor–treated mice. **(C)** Tumor weight was measured and compared at the last day of the experiment (mean ± SD). **(D)** Expression of BAK, P21, cleaved caspase-3, p-SHP2, SHP2, p-ERK, and ERK in different groups was measured by Western blotting and was then quantified by ImageJ software; GAPDH was used as a loading control. Data shown are mean ± SD. **p < 0.01 and ***p < 0.001.

Having shown that SHP2 inhibitors induced cell death *in vitro*, we further evaluated the anti-myeloma efficacy of SHP2 inhibitors *in vivo* by using MM murine xenograft models developed by subcutaneous injection of RPMI-8226 cells into the left flank of the Balb/c nude mice. Oral administration of SHP2 inhibitors (SHP099, 75 mg/kg; RMC-4550, 30 mg/kg) or vehicle control was carried out at daily doses when tumors became palpable. As shown in Figures 5A–C, tumor size, growth, and weight in MM xenograft mice reduced with the administration of SHP099 and RMC-4550 compared with the vehicle-treated xenograft mice. Moreover, tumor masses from the mice treated with SHP2 inhibitors exhibited distinct tumor mesenchyme and reduced pathological mitosis *via* H&E staining. In addition, the expression of BAK, P21, and cleaved caspase-3 in xenografted tumors was considerably increased, while the level of p-SHP2 and p-ERK was decreased upon SHP2 inhibitor treatment (Figure 5D). These results indicated that both SHP099 and RMC-4550 could inhibit the xenografted myeloma tumor growth in mice.

### 3.5 SHP2 Inhibitors in Combination With BTZ Shows a Superior Synergistic Cytotoxicity Against MM Cells

**FIGURE 6:**
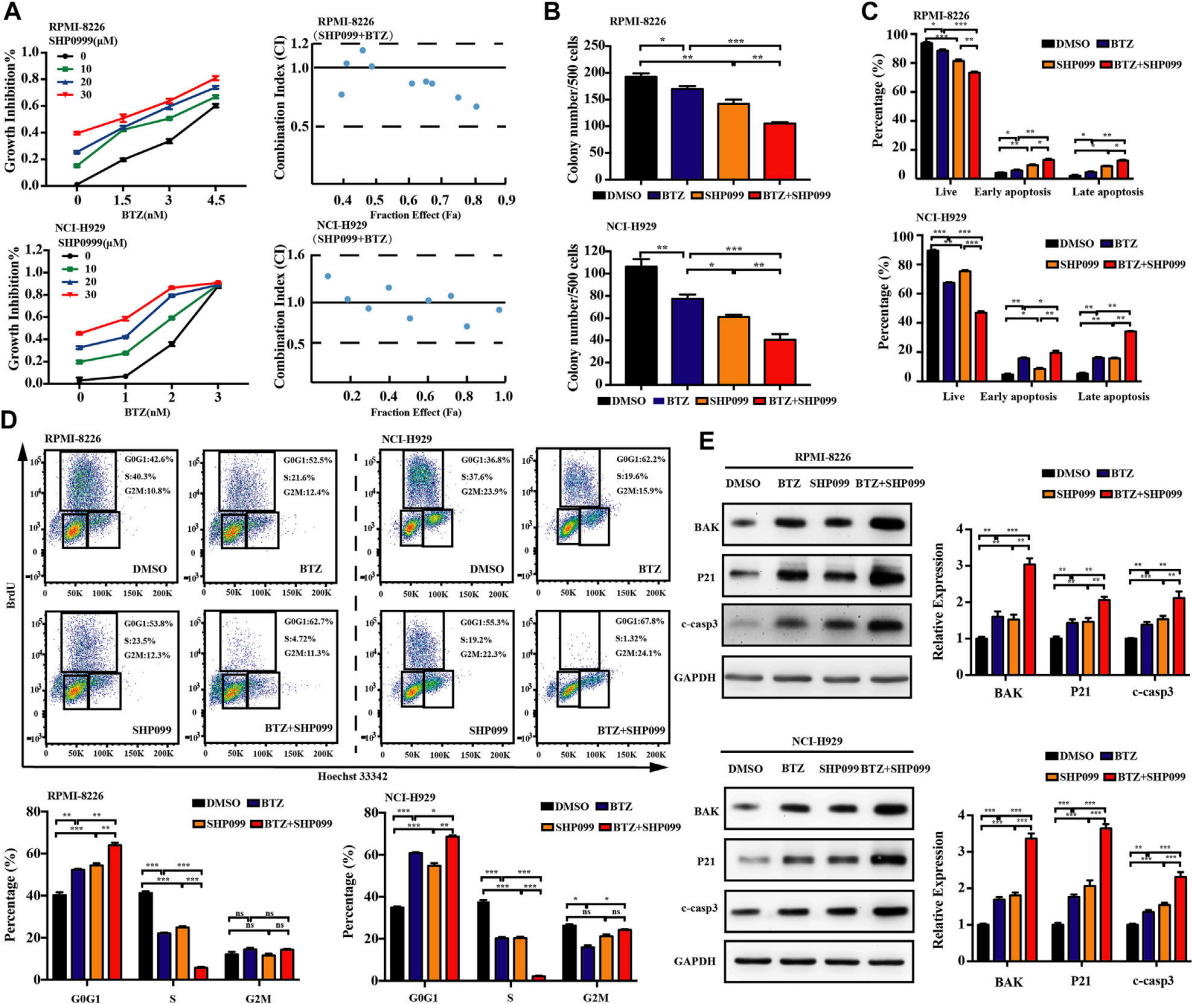
Co-treatment of SHP099 and BTZ is synergistically cytotoxic to BTZ-sensitive MM cells. **(A)** Effect of treatment with different concentrations of BTZ alone or in combination with different doses of SHP099 for 48 h on cell viability of two MM cell lines, RPMI-8226 and NCI-H929, was measured using CCK-8 assay. Combination index (CI) for two drugs in the MM cell lines was shown. x-axis, fractional effect concentrations; y-axis, CI. RPMI-8226 and NCI-H929 cells were treated with DMSO, SHP099 (20 μM), BTZ (3 nM for RPMI-8226 and 2 nM for NCI-H929 cells respectively), or SHP099 plus BTZ for 48 h, and then colony formation **(B)**, cell apoptosis **(C),** and cell cycle **(D)** were detected. **(E)** Immunoblot analysis of RPMI-8226 and NCI-H929 cell lines treated with SHP099 (20 μM), BTZ (3 nM for RPMI-8226 and 2 nM for NCI-H929 cells), or SHP099 plus BTZ for 48 h; the relative amount of the protein level, normalized to that of GAPDH expression, was analyzed by ImageJ software. Data were presented as mean ± SD from three independent experiments; *p < 0 .05, **p < 0.01, and ***p < 0.001.

**FIGURE 7:**
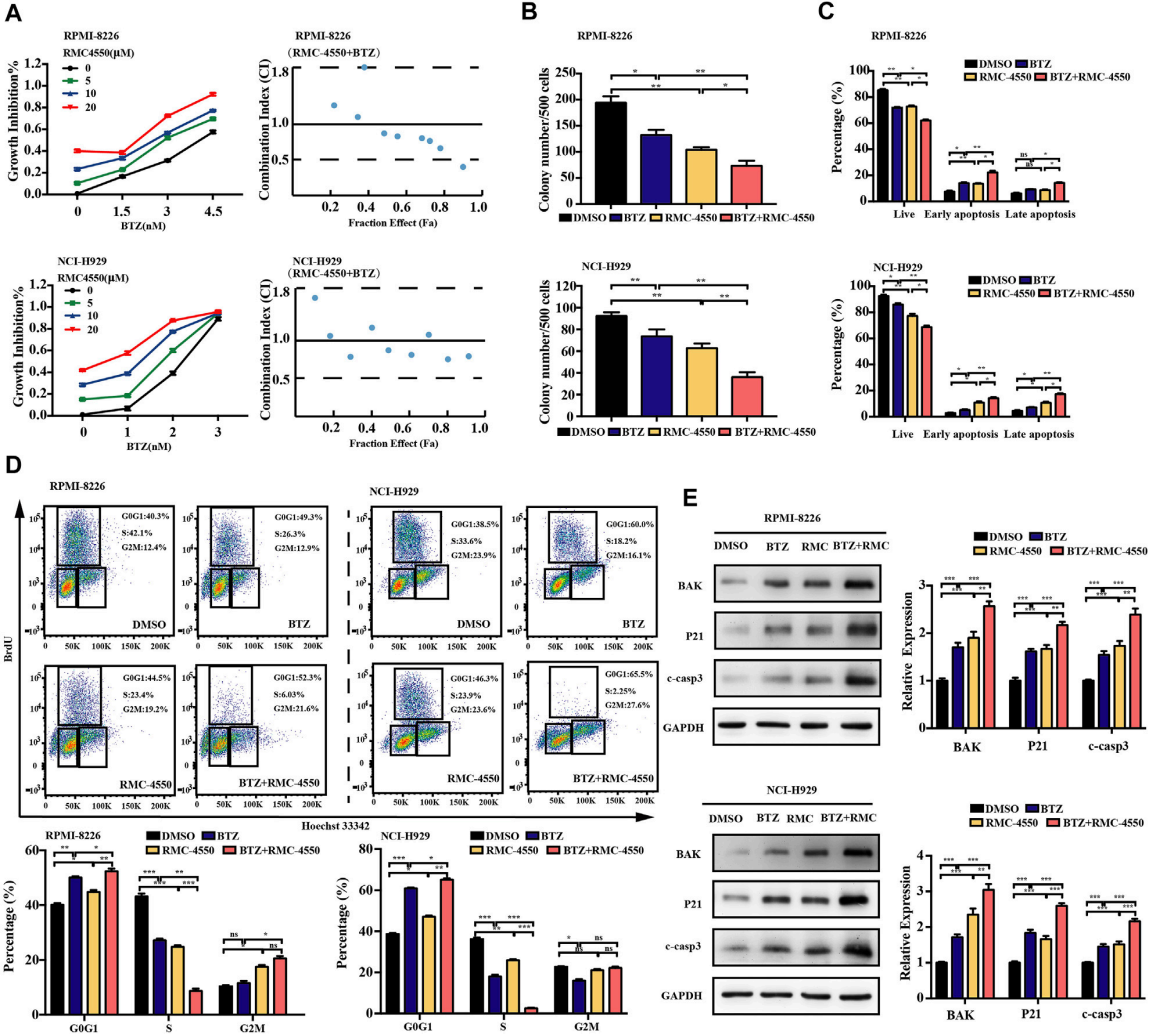
Combination of RMC-4550 and BTZ exhibits a synergistic antitumor effect against MM cells. **(A)** Cytotoxic effects of various doses of RMC-4550 and BTZ in monotherapy and in combination on MM cells determined by the CCK-8 assay. Fa-CI plot analysis of combination treatment of the two on RPMI-8226 and NCI-H929 cell viability was performed. x-axis, fractional effect concentrations; y-axis, CI. RPMI-8226 and NCI-H929 cells were culture in the presence of DMSO, RMC-4550 (10 μM), BTZ (3 nM for RPMI-8226 and 2 nM for NCI-H929 cells), or RMC-4550 plus BTZ for 48 h, and then colony formation **(B)**, cell apoptosis **(C),** and cell cycle **(D)** were measured. The representative flow cytometric profiles of BrdU/Hoechst 33342 are shown. **(E)** Western blotting shows the levels of P21, BAK, and cleaved caspase-3 in myeloma cells with monotherapy or the combination of bortezomib and RMC4550 for 48 h. GAPDH served as a loading control. The graphs show the density volumes of the indicated proteins normalized to those in the control. Data were displayed as mean ± SD from three independent experiments. *p < 0.05, **p < 0.01, and ***p < 0.001.

Although BTZ is successfully applied in the treatment of MM for decades, its efficacy is still restricted by the occurrence of resistance. The combination of BTZ with other novel therapeutic agents may enhance its therapeutic effect. To determine whether combined exposure to bortezomib and SHP2 inhibitors would have a synergistic effect on the survival of myeloma cells, RPMI-8226 and NCI-H929 cells were cultured with different doses of bortezomib or SHP2 inhibitors alone or in combination for 48 h and the CCK-8 assay was measured. The antagonism or synergism of the combinations was evaluated using Chou–Talalay analysis. As shown in Figure 6A, SHP099 plus BTZ was synergistic in most dose combinations but was additive or antagonistic in some dose combinations in both MM cells. Specifically, when BTZ at the concentrations of 3.0 and 4.5 nM combined with any tested doses of SHP099 (10, 20, and 30 μM), synergistic effects were observed in RPMI-8226 cells. In the NCI-H929 cell line, the combination of BTZ at a dose of 2 nM with 20 or 30 μM SHP099 and 3 nM BTZ with 10 or 30 μM SHP099 had synergistic cytotoxicities. (Figure 6A; Supplementary Table S1). In addition, we also found that treatment of MM cells with the SHP099/BTZ combination (BTZ 3 nM for RPMI-8226 or 2 nM BTZ for NCI-H929 combined with 20 μM SHP099) was associated with inferior capability of colony formation than that observed with SHP099 or BTZ alone (Figure 6B). Moreover, SHP099 and BTZ co-treated MM cells showed significant increase in the percentage of apoptotic cells (Figure 6C). To further investigate the role of cell cycle arrest induced by SHP099 plus BTZ, we used BrdU staining assay and found that the co-treatment with SHP099 and BTZ synergistically blocked the G1/S phase cell cycle transition (Figure 6D). In addition, the expression of BAK, cleaved caspase-3, and P21 increased in RPMI-8226 and NCI-H929 cells treated with the combination of SHP099 and BTZ (Figure 6E). Consistently, similar results were observed in RMC-4550 and BTZ co-treated RPMI-8226 and NCI-H929 cells (Figures 7A–E; Supplementary Table S2).

### 3.6 SHP2 Inhibitors Overcome Bortezomib Resistance in Human MM Cells

**FIGURE 8:**
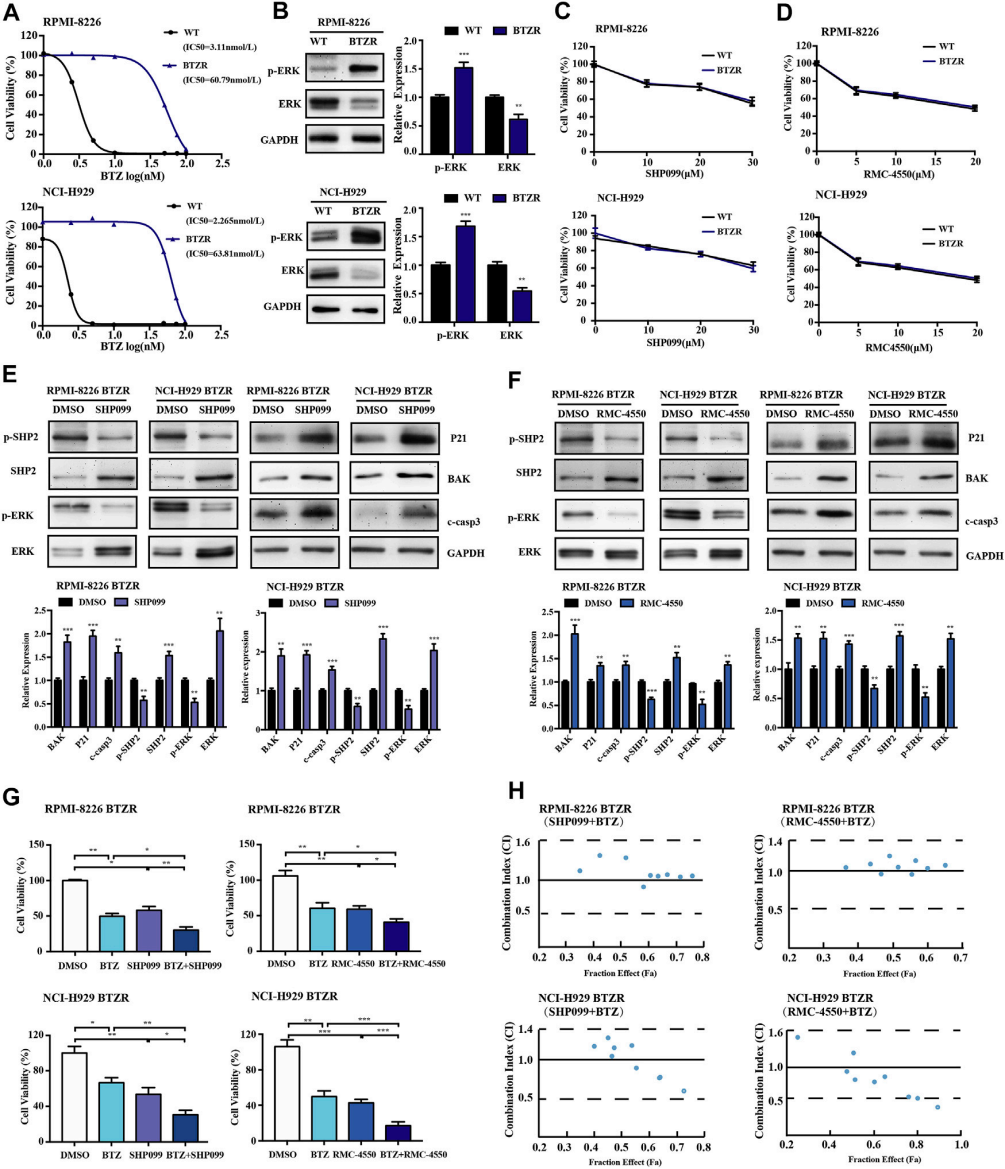
Pharmacological inhibition of SHP2 with SHP099 or RMC-4550 is effective in suppressing the growth and overcoming bortezomib resistance in bortezomib-resistant MM cells. **(A)** Bortezomib naïve (WT) and resistant (BTZR) myeloma cells (RPMI-8226 and NCI-H929 cells) were treated with various concentrations of bortezomib for 48 h, and the IC_50_ was calculated and shown. **(B)** Protein levels of p-ERK and ERK of the parental and bortezomib-resistant myeloma cells were detected by Western blot analysis. The bar graph illustrates the quantitative comparison of different proteins levels. **(C,D)** Pharmacological inhibition of SHP2 with SHP099 or RMC-4550 in bortezomib-resistant myeloma cells (BTZR) conferred a similar growth inhibition effect to that of bortezomib-naïve myeloma cells (WT). **(E,F)** After incubation with 20 µM SHP099 or 10 µM RMC-4550 in RPMI-8226 and NCI-H929 cells for 48 h, Western blots against p-SHP2, SHP2, p-ERK, ERK, P21, BAK, and cleaved caspase-3 were performed and quantified. **(G)** Bortezomib-resistant (RPMI-8226 BTZR or NCI-H929 BTZR) cells were treated with either bortezomib (50 nM), SHP2 inhibitors (20 μM SHP099 or 10 μM RMC-4550), or the combination for 48 h, followed by assessment for cell viability. **(H)** Bortezomib-resistant MM cells were incubated with different doses of BTZ and SHP2 inhibitors (SHP099 or RMC-4550) or with the combination of both for 48 h; the synergistic cytotoxic effects were determined using the combination index based on the data from cell viability assays. x-axis, fractional effect concentrations; y-axis, CI. Data were displayed as mean ± SD from three independent experiments. *p < 0.05, **p < 0.01, and ***p < 0.001.

Given the antitumor activity of SHP2 inhibitors in BTZ-sensitive MM cells, we tried to investigate whether SHP2 inhibitors have an effect on BTZ-resistant MM cells. Herein, we cultured RPMI-8226 and NCI-H929 over a period of 1 year with gradually increased concentrations of BTZ and successfully generated BTZ-resistant MM cell lines. Compared to their parental BTZ-naïve cells, the IC_50_ of the BTZ-resistant myeloma cell lines to BTZ has increased by about 20 folds (Figure 8A). Moreover, we verified that bortezomib-resistant cells have a higher level of p-ERK in comparison to the naïve myeloma cells (Figure 8B). However, our results showed a decrease in the levels of p-SHP2 and SHP2 in bortezomib-resistant MM cells relative to bortezomib-naïve MM cells (Supplementary Figure S1). Next, we tried whether bortezomib-resistant cells with enhanced ERK activation could be sensitive to SHP2 inhibitors. Then, BTZ naïve and resistant myeloma cell lines were treated with SHP099 or RMC-4550 for 48 h, and we found that SHP2 inhibitors were effective despite a relatively lower level of p-SHP2 in BTZ-resistant MM cells than that in BTZ naïve MM cells (Figure 8C,D). Furthermore, we observed that both two SHP2 inhibitors treatment decreased p-SHP2 and p-ERK protein levels, while they induced protein levels of SHP2, ERK, P21, BAK, and cleaved caspase-3 in BTZ-resistant myeloma cells (Figures 8E,F). When we challenged BTZ-resistant MM cells with bortezomib (50 nM) in combination with SHP099 (20 μM) or RMC-4550 (10 μM), we also observed a great decrease in cell viability of BTZ-resistant MM cells receiving combination therapy (Figures 8G). In order to determine whether combination therapy maintains its synergistic effect in BTZ-resistant MM cells, we used the Chou–Talalay method. Unexpectedly, only additive or antagonistic effects were observed in BTZ and SHP2 inhibitor combined therapy applied to RPMI-8226 BTZR cells. Nevertheless, both the SHP2 inhibitors were synergistic with BTZ in NCI-H929 BTZR cells. The combination of 45 or 50 nM BTZ with SHP099 (20 or 30 μM) and the combination of 45 or 50 nM BTZ plus all tested doses of RMC-4550 (5, 10, and 20 μM) triggered the synergistic antitumor activity in NCI-H929 BTZR cells (Figure 8H; Supplementary Tables S3, S4). Collectively, these findings suggested that SHP2 inhibitors could overcome bortezomib resistance of human MM cells.

## 4 Discussion

Despite the tremendous revolution in therapeutic strategies in the era of novel agents, the management of MM remains challenging due to the inevitable relapse or refractory nature, and the advent of novel therapies is, therefore, awaited. The activation of signaling cascades including RAS/RAF/MEK/ERK, PI3K/AKT, NF-κB, and JAK/STAT pathway is considered the main mechanism in the development of MM (Hu and Hu, 2018). Specifically, mutation clusters in RAS/RAF genes are as high as 50% in newly diagnosed MM, rising even to 80% in RRMM, underscoring the promising potential of the RAS/RAF/MEK/ERK signaling pathway–directed therapy (Podar and Leleu, 2021). Unfortunately, treatments targeting the RAS or its downstream/upstream effectors, such as tipifarnib and sorafenib, show limited/no activity in MM (Lind et al., 2019).

As reported, SHP2 is indispensable for the activation of the RAS/ERK pathway and might play an oncogenic role in MM for the reason that SHP2 mediates the protective effect of interleukin-6 against dexamethasone-induced apoptosis in MM cells, and its level is associated with poor response to induction therapy and prognosis of MM (Chauhan et al., 2000; Beldi-Ferchiou et al., 2017). To date, several allosteric SHP2 inhibitors with high bioactivity and selectivity have successfully entered into clinical trials for mono- or combined therapy of cancers (Wu et al., 2021). However, the therapeutic value of SHP2 inhibitors for MM remains undetermined. In the current study, we used two allosteric inhibitors of SHP2 (SHP099 and RMC-4550) and found that both the SHP2 inhibitors could inhibit MM cell proliferation and suppress SHP2 phosphorylation in a concentration-dependent manner, demonstrating the common anti-myeloma activity of SHP2 inhibitors. Specifically, SHP2 inhibitors induced apoptosis and cell-cycle arrest of MM cells. The evidence for apoptosis induction of SHP2 inhibitors was revealed by increased Annexin-V positive cells, cleavage of caspase-3, and increased level of BAK in MM cells. In addition, SHP2 inhibitors could induce cell-cycle arrest at the G1/S phase *via* transcriptional activation of P21. Furthermore, using murine xenograft models, we found that SHP2 inhibitors attenuated progression of myeloma *in vivo*. Therefore, blocking the activation of SHP2 by specific inhibitors is an attractive strategy for targeting MM.

As we know, activating Ras mutations, which have a high incidence varying between 32% and 50% in MM and were featured with greater tumor burden, emphasizes the potential significance of RAS/RAF/MEK/ERK signaling as a therapeutic target (Chang-Yew Leow et al., 2013). Notably, an increasing number of research studies have indicated that SHP2 inhibitors show efficacy in subsets of RTK-, KRAS-, and BRAF-driven cancers, although it has been previously reported that cancer cells carrying oncogenic RAS/RAF mutations would be refractory to SHP2 inhibition (Chen et al., 2016; Kerr et al., 2021). However, cancer cells bearing distinct RAS mutations exhibit variable sensitivity to SHP2 inhibitors (Gebregiworgis et al., 2021). In this study, our results suggested that both the myeloma cell lines, 8226 (KRAS G12A) and H929 (NRAS G13D), are sensitive to SHP2 inhibitors, the latter of which contradicts previous reports that RAS G13D–mutated cancer cells are resistant to SHP2 inhibition (Nichols et al., 2018). The underlying mechanisms of the resistance to SHP2 inhibitors in cells harboring KRAS G13D are that RAS (G13D) activation of RAS is not dependent on upstream RTK/SHP2 signaling and is characterized by a high rate of nucleotide exchange and very low intrinsic GTPase activity (Ahmed et al., 2019). This discrepancy of the data was brought to our attention. In fact, the effectiveness of SHP inhibitors on KRAS G13D–mutated tumor cells has also been found in other studies. For example, the KRAS G13D–mutated breast cancer cell line (MDA-MB-231) and colorectal cancer cell line (NCI-H747) were sensitive to the SHP2 inhibitors SHP099 and TNO155, respectively (Ahmed et al., 2019; Liu et al., 2021). Gebregiworgis T et al. showed that cells harboring KRAS Q61H are uniquely resistant to SHP2 inhibitors and elucidated that Q61H mutation is insensitive to SOS1-mediated nucleotide exchange and its GTPase cycle is severely decoupled from regulation by the GEF and GAP activities of SOS1 and RASA1. However, we noticed that KRAS G13D exhibited a strong increase in the nucleotide-exchange rate in response to SOS1-assisted nucleotide-exchange kinetics, and the rate of GAP-stimulated GTP hydrolysis in G13D mutation was higher than that in Q61H mutation (Hunter et al., 2015; Gebregiworgis et al., 2021). In addition, the sensitivity to SHP099 was positively correlated with the basal level of RAS activity in SHP099-sensitive KRAS mutant cell lines. Morgan MA et al. have demonstrated that both RPMI-8226 and NCI-H929, which contain RAS mutations, exhibit high levels of activated RAS by activated RAS-binding assays and MEK/MAPK activation by Western blot analysis (Morgan et al., 2005). Therefore, we hypothesize that the sensitivity of KRAS G13D mutant cancer cells to SHP2 inhibitors might be associated with the basal RAS activity or MEK/MAPK activation, the response to the upstream regulator–mediated nucleotide exchange and the rate of GAP-stimulated GTP hydrolysis of cancer cells.

It has been shown that ERK phosphorylation catalyzed by activated MAPK/ERK kinases (MEK) downstream of the Ras proto-oncogene transduces the activation of the MAPK/ERK pathway, playing a critical role in controlling cell proliferation, apoptosis, cell cycle, and drug resistance in MM. Particularly, SHP2 acts as a key scaffold protein recruiting the GRB2/SOS complex and activates the RAS/ERK signal transduction pathway in many malignancies, including MM (Rehman et al., 2018; Ishibashi et al., 2020). Pharmacologic inhibition of SHP2, including SHP099 and RMC-4550, could suppress the growth of various tumors harboring KRAS mutations, such as pancreatic and lung cancers, *via* regulating RAS/MAPK signaling (Nichols et al., 2018; Hao et al., 2019). Therefore, to elucidate the molecular mechanism of SHP2 inhibitor–induced apoptosis and cell cycle arrest in the MM cells, we subsequently evaluated the activation status of MAPK signaling. In our study, we demonstrated that both the allosteric SHP2 inhibitors (SHP099 and RMC-4550) inhibited the phosphorylation of SHP2 at Y542 and blocked the activation of ERK in a dose-dependent manner. However, SHP2 inhibitors exhibited no impact on the phosphorylation of AKT in MM cells (data not shown). These results show that SHP2 inhibitors could be promising Raf/MEK/ERK signaling inhibitors for the treatment of MM without affecting the PI3K-AKT pathway.

Despite the success of BTZ in the treatment of MM in real-life practice, acquired resistance of BTZ is still a major clinical obstacle for relapsed and refractory MM patients (Zhang et al., 2021). Since the combination therapy with BTZ is a good choice to avoid drug resistance than monotherapy, we combined SHP2 inhibitors with BTZ and found synergistic antitumor activity in MM cells with enhanced expression of P21, BAK, and cleaved caspase-3. In addition, looking for novel therapeutic agents is an alternative to overcome bortezomib resistance. It is worth mentioning that previous reports and our research confirmed that BTZ-resistant MM cells have an enhanced activation of the MAPK/ERK pathway (Lee et al., 2021). Thus, we subsequently analyzed whether SHP2 inhibitors could be effective against BTZ-resistant MM cells. The results of our study illustrated that SHP2 inhibitors exhibited comparable cytotoxic activity against BTZ-resistant MM cells compared to BTZ-naïve MM cells by downregulating the phosphorylation of ERK. Then, we performed the Chou–Talalay method to determine whether the combination of SHP2 inhibitors and BTZ had additive or synergistic cytotoxicity in BTZ-resistant MM cells. However, index calculations showed that SHP2 inhibitors with BTZ had an additive or antagonistic effect on RPMI-8226 BTZR cells, while its combination had a synergistic effect on NCI-H929 BTZR cell survival. These data supported that SHP2 inhibitors might be promising candidates for MM patients resistant to bortezomib chemotherapy and the combination of SHP2 inhibitors and BTZ might be a good choice to overcome the resistance of BTZ.

However, there are some limitations in our study. For example, since we just tested the therapeutic effects of SHP2 inhibitors in RPMI-8226 and NCI-H929 cells, which express activated N-Ras or K-Ras (Bolick et al., 2003), further efforts should be applied to compare the efficacy of the SHP2 inhibitors in Ras wild-type vs. mutant MM cell lines in future. A previous report has reported that PRL3, a phosphatase that inhibits the activation of SHP2, was able to promote the resistance of MM cells to BTZ (Chong et al., 2019). Another study has indicated that bortezomib treatment does not alter any changes in the protein level of SHP2 (Chauhan et al., 2004). In our study, we found that changes in the level of p-SHP2 were inconsistent with the level of p-ERK in BTZ-resistant MM cells. Thus, we speculated that the role of SHP2 in the acquisition of BTZ resistance in MM is complex and needs to be explored further.

## 5 Conclusion

In summary, our study shows that SHP2 allosteric inhibitors could suppress the survival of MM cells by inducing cell apoptosis and cell cycle arrest. As per the mechanism, reduced activation of ERK may be responsible for the anti-myeloma effect of SHP2 inhibitors. The combination of SHP2 inhibitors with BTZ induces synergistic cytotoxicity in BTZ naïve MM cells. Moreover, SHP2 inhibitors are active against BTZ-resistant MM cells. Thus, these findings imply that SHP2 inhibitors may become attractive therapeutic agents as either monotherapy or combination therapy for MM in the future.

## Data Availability

The raw data supporting the conclusion of this article will be made available by the authors, without undue reservation.
