# Supplementary material for: SHP2 Inhibitors Show Anti-Myeloma Activity and Synergize With Bortezomib in the Treatment of Multiple Myeloma
Source: Front Pharmacol. 2022 Apr 6;13:841308. doi: 10.3389/fphar.2022.841308 (PMC9019471; doi:10.3389/fphar.2022.841308)
Supplement: Supplementary file 1 [file DataSheet1.pdf]

SHP2 inhibitors show anti-myeloma activity and synergize with bortezomib in the treatment of multiple myeloma.

Pan Zhou[1], Mengyu Xiao[1], Weiya Li[1], Xiaobai Sun[1], Yanliang Bai[1], Feiying Meng[2], Zunmin Zhu[1], Weiping Yuan[3]*, Kai Sun[1]*

[1.]Department of Hematology, Zhengzhou University People's Hospital, Henan Provincial People's Hospital, Zhengzhou, China.

[2.]Henan Eye Institute, Henan Eye Hospital, Henan Provincial People's Hospital, Zhengzhou University People's Hospital，Zhengzhou, China.

[3.]State Key Laboratory of Experimental Hematology, Institute of Hematology and Blood Diseases Hospital, Chinese Academy of Medical Sciences and Peking Union Medical College, Tianjin, China.

Supplementary Table 1. Concentration-dependent combined effects of SHP099 and BTZ in RPMI-8226 and NCI-H929 cell lines. Fa, fraction affected; CI, combination index. CI of < 1 represents synergism, CI of 1 or close to 1 represents additive effects, and CI of > 1 represents antagonism.

Dose SHP099 (μM)	Dose BTZ (nM)	RPMI-8226		Dose SHP099 (μM)	Dose BTZ (nM)	NCI-H929	
		Fa	CI			Fa	CI
10	1.5	0.41243	0.77605	10	1	0.26620	1.33957
10	3.0	0.49798	0.99333	10	2	0.59157	1.09967
10	4.5	0.66667	0.86517	10	3	0.89352	0.9696
20	1.5	0.42615	1.01876	20	1	0.41731	1.25532
20	3.0	0.61009	0.86352	20	2	0.79685	0.86943
20	4.5	0.74288	0.75553	20	3	0.88657	1.01853
30	1.5	0.47054	1.11706	30	1	0.56713	1.05478
30	3.0	0.65066	0.87882	30	2	0.85648	0.80125
30	4.5	0.79177	0.68602	30	3	0.90509	0.95184

Supplementary Table 2. Concentration-dependent combined effects of RMC-4550 and BTZ in RPMI-8226 and NCI-H929 cell lines. Fa, fraction affected; CI, combination index. CI of < 1 represents synergism, CI of 1 or close to 1 represents additive effects, and CI of > 1 represents antagonism.

Dose RMC-4550 (μM)	Dose BTZ (nM)	RPMI-8226		Dose RMC-4550 (μM)	Dose BTZ (nM)	NCI-H929	
		Fa	CI			Fa	CI
5	1.5	0.22357	1.25193	5	1	0.17361	1.66691
5	3.0	0.51009	0.86895	5	2	0.59491	1.10389
5	4.5	0.68765	0.79127	5	3	0.95139	0.78443
10	1.5	0.33656	1.0939	10	1	0.37963	1.23145
10	3.0	0.56765	0.84983	10	2	0.77315	0.90896
10	4.5	0.76998	0.66291	10	3	0.9375	0.85371
20	1.5	0.3753	1.78113	20	1	0.56019	1.12335
20	3.0	0.71421	0.75869	20	2	0.86806	0.79924
20	4.5	0.9088	0.40428	20	3	0.94676	0.84014

Supplementary Figure 1. Decreased p-SHP2 and SHP2 expression in bortezomib resistant myeloma cell lines. (A) Images show the most representative blots of p-SHP2 and SHP2 in bortezomib-resistant RPMI-8226 and NCI-H929 cells (BTZR). Bortezomib naïve MM cells (WT) were used as controls and GAPDH was used as loading control. **(B)** The graph represents the relative quantification for indicated proteins expression as mean ± SD of three independent experiments. * $p < 0.05$ and ** $p < 0.01$.

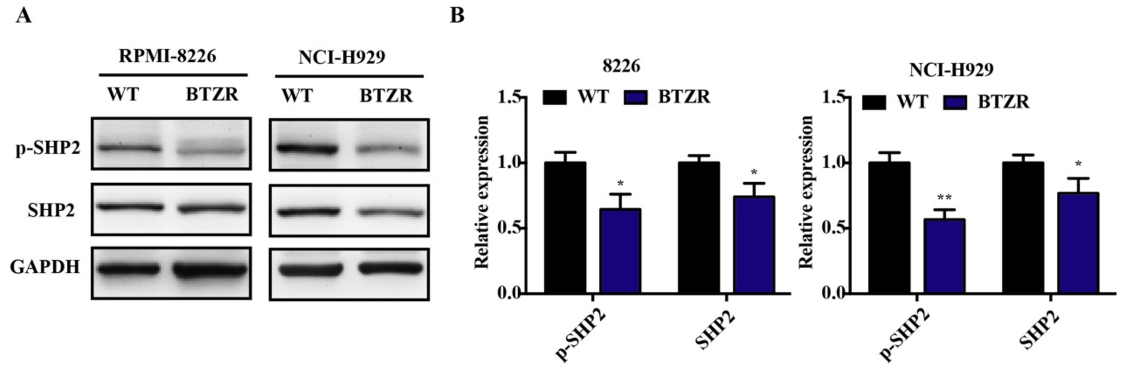

Supplementary Table 3. Combination index of bortezomib combination treatment with SHP099 in bortezomib resistant cell lines (RPMI-8226 BTZR and NCI-H929 BTZR). Fa, fraction affected; CI, combination index. CI of < 1 represents synergism, CI of 1 or close to 1 represents additive effects, and CI of > 1 represents antagonism.

Dose SHP099 (μM)	Dose BTZ (nM)	RPMI-8226 BTZR		Dose SHP099 (μM)	Dose BTZ (nM)	NCI-H929 BTZR	
		Fa	CI			Fa	CI
10	40	0.37196	1.15501	10	40	0.40988	1.16467
10	45	0.58104	0.93948	10	45	0.46802	1.04129
10	50	0.63128	1.07786	10	50	0.47674	1.14822
20	40	0.43841	1.36703	20	40	0.45640	1.36182
20	45	0.60535	1.09011	20	45	0.56395	0.92289
20	50	0.70421	1.06299	20	50	0.63953	0.76677
30	40	0.52512	1.33988	30	40	0.53488	1.16716
30	45	0.65964	1.10873	30	45	0.64244	0.77337
30	50	0.73906	1.08169	30	50	0.72093	0.613660

Supplementary Table 4. Combination index of bortezomib combination treatment with RMC-4550 in bortezomib resistant cell lines (RPMI-8226 BTZR and NCI-H929 BTZR). Fa, fraction affected; CI, combination index. CI of < 1 represents synergism, CI of 1 or close to 1 represents additive effects, and CI of > 1 represents antagonism.

Dose RMC-4550 (μM)	Dose BTZ (nM)	RPMI-8226 BTZR		Dose RMC-4550 (μM)	Dose BTZ (nM)	NCI-H929 BTZR	
		Fa	CI			Fa	CI
5	40	0.36277	1.18783	5	40	0.28218	1.43393
5	45	0.43377	1.18505	5	45	0.52228	0.85041
5	50	0.48918	1.29082	5	50	0.74752	0.60653
10	40	0.4658	0.96395	10	40	0.4901	0.95428
10	45	0.55325	0.96167	10	45	0.60396	0.81445
10	50	0.61268	1.07063	10	50	0.78465	0.58571
20	40	0.52294	1.06275	20	40	0.51733	1.22027
20	45	0.5645	1.13654	20	45	0.64851	0.89139
20	50	0.65385	1.09704	20	50	0.86881	0.47022
